# A Classification Algorithm-Based Hybrid Diabetes Prediction Model

**DOI:** 10.3389/fpubh.2022.829519

**Published:** 2022-03-31

**Authors:** Michael Onyema Edeh, Osamah Ibrahim Khalaf, Carlos Andrés Tavera, Sofiane Tayeb, Samir Ghouali, Ghaida Muttashar Abdulsahib, Nneka Ernestina Richard-Nnabu, AbdRahmane Louni

**Affiliations:** ^1^Department of Mathematics and Computer Science, Coal City University, Enugu, Nigeria; ^2^Al-Nahrain Nanorenewable Energy Research Center, Al-Nahrain University, Baghdad, Iraq; ^3^COMBA R&D Laboratory, Faculty of Engineering, Universidad Santiago de Cali, Cali, Colombia; ^4^Department of Electrical Engineering, Faculty of Sciences and Technology, Mustapha Stambouli University of Mascara, Mascara, Algeria; ^5^Department of Electrical Engineering, Faculty of Science and Technology, University of Mascara, Mascara, Algeria; ^6^STIC Laboratory, Faculty of Engineering Science, Tlemcen, Algeria; ^7^Department of Computer Engineering, University of Technology, Baghdad, Iraq; ^8^Department of Computer Science/Informatics, Alex Ekwueme Federal University Ndufu Alike Ikwo (AE-FUNAI), Abakaliki, Nigeria; ^9^Faculty of Sciences and Technology, Mustapha Stambouli University of Mascara, Mascara, Algeria

**Keywords:** decision tree, random forest, Support Vector Machine (SVM), Bayesian Naive, diabetes, AI, ML, classification

## Abstract

Diabetes is considered to be one of the leading causes of death globally. If diabetes is not treated and detected early, it can lead to a variety of complications. The aim of this study was to develop a model that can accurately predict the likelihood of developing diabetes in patients with the greatest amount of precision. Classification algorithms are widely used in the medical field to classify data into different categories based on some criteria that are relatively restrictive to the individual classifier, Therefore, four machine learning classification algorithms, namely supervised learning algorithms (Random forest, SVM and Naïve Bayes, Decision Tree DT) and unsupervised learning algorithm (k-means), have been a technique that was utilized in this investigation to identify diabetes in its early stages. The experiments are per-formed on two databases, one extracted from the Frankfurt Hospital in Germany and the other from the database. PIMA Indian Diabetes (PIDD) provided by the UCI machine learning repository. The results obtained from the database extracted from Frankfurt Hospital, Germany, showed that the random forest algorithm outperformed with the highest accuracy of 97.6%, and the results obtained from the Pima Indian database showed that the SVM algorithm outperformed with the highest accuracy of 83.1% compared to other algorithms. The validity of these results is confirmed by the process of separating the data set into two parts: a training set and a test set, which is described below. The training set is used to develop the model's capabilities. The test set is used to put the model through its paces and determine its correctness.

## Introduction

Diabetes is a chronic disease also known as a silent disease. The World Health Organization (WHO) defines diabetes as a disease that prevents the body from properly using the energy provided by the food it consumes. In addition, the disease occurs when there are problems with the hormone insulin, which is naturally produced by the pancreas to help the body use sugar and fat and store some it (1).

More clearly, when we eat, food is broken down into glucose (sugar). This glucose provides energy for the body to function properly by drawing on its resources. During digestion, the blood carries the glucose throughout the body and supplies the cells. However, in order for the sugar in the blood to be delivered to the cells, the body needs insulin, a hormone secreted by the pancreas, which acts as a key to get the glucose from the blood into the cells of our body (2). There are three most common types of diabetes:

Type 1 diabetes: it is a condition in which the pancreas cannot produces enough insulin or does nor produces any insulin. It accounts for 5–10% of all diabetes cases, commonly affecting childhood and adolescence. Then, Type 2 diabetes–it is a condition in which the insulin produced does not effectively used to maintain the blood sugar level in the body. This type diabetes is common in people age 40 and above, but also appears in younger people. From all diagnosed diabetes cases worldwide, type 2 diabetes accounts for 90–95 percent. There is another type of diabetes called “gestational diabetes”, caused by the lack response of the insulin receptors on the body tissues, even if the insulin levels are normal, which makes this condition different from the second type, and this case is very rare, account for 1–2% of all diabetes cased and it also increase the risk of developing type 2 diabetes later (3).

People with diabetes must be treated according to their type of diabetes. The goal of treatment is to keep the patient's blood sugar level within a normal range.

## Related Works

**Table:** 

**Authors**	**Study abstract**	**Reference**
Kumari and Chitra	In the proposed work, SVM with radial basis function kernel is used for classification. The performance parameters such as the classification accuracy (78.2 %), sensitivity (80%), and specificity of the SVM and RBF have found to be high thus making it a good option for the classification process.	(4)
Ahmed	Accuracy of the proposed models has been compared. The random forest method provided an accuracy of 74.7%, ANN gave 75.7% and K-means clustering method has given 73.6% accuracy.	(5)
Shetty et al.	Used K-Nearest Neighbors (KNN) and the Naïve Bayes technique for diabetes prediction. This technique was implemented in the form of a software program, in which users provide data in terms of patient records and the finding that the patient is diabetic or not.	(6)
Bhoia et al.	In this paper. Various supervised learning algorithms have been used such as CT, SVM, k-NN, NB, RF, NN, AB, and LR, and generated the training dataset	(7)
	and testing dataset using k-fold cross-validation with k = 10. The results of accuracy = 76.80%	
Kandhasamy and Balamurali	The authors in used data from the University of California, diabetes mellitus patients were classified using a machine learning data repository to compare the performance of four common classifiers (J48 DT, the K-Nearest Neighbors algorithm, the Random Forest algorithm, and the Support Vector Machines algorithm). They used a data sample from the UCI machine learning data repository. Preliminary results suggest that the J48 DT classifier outperforms the other three classifiers in terms of accuracy (73.82 percent) before data preparation, and that the KNN (k = 1) and Random Forest classifiers outperform the other three classifiers after data pre-processing.	(8)
Vijayan et al.	The KNN method and the ANFIS algorithm are comparable. According to the results of the experiment, the amalgam of KNN and ANFIS gives the highest classification accuracy of 80 % among the algorithms tested.	(9)
Soleh et al.	The data in this study divided into two, 75% for training data, and 25% for testing data. This study produces an evaluation with an accuracy 80%, which means it is better than the previous paper, which is 75, 97%.	(10)
Rajput et al.	The target of analysis made in the present research is to list the risks factors and correlation that exist among those risk factors. In this work, logistic regression, support vector machine, random forest, decision tree, Naive Bayes, K nearest neighbor classifiers are used for prediction, and their accuracy is compared to choose the better machine learning model. SVM provides higher accuracy (96.0) among the choosen algorithms.	(11)
Deepa et al.	This work aims to propose an artificial intelligence-based intelligent system for earlier prediction of the disease using Ridge-Adaline Stochastic Gradient Descent Classifier (RASGD. The results of the proposed scheme have been compared with state-of-the-art machine learning algorithms such as support vector machine and logistic regression methods. The RASGD intelligent system attains an accuracy of 92%, which is better than the other selected classifiers.	(12)

## Methodology Used

### Model Diagram and Study Explanation

**Figure 1:**
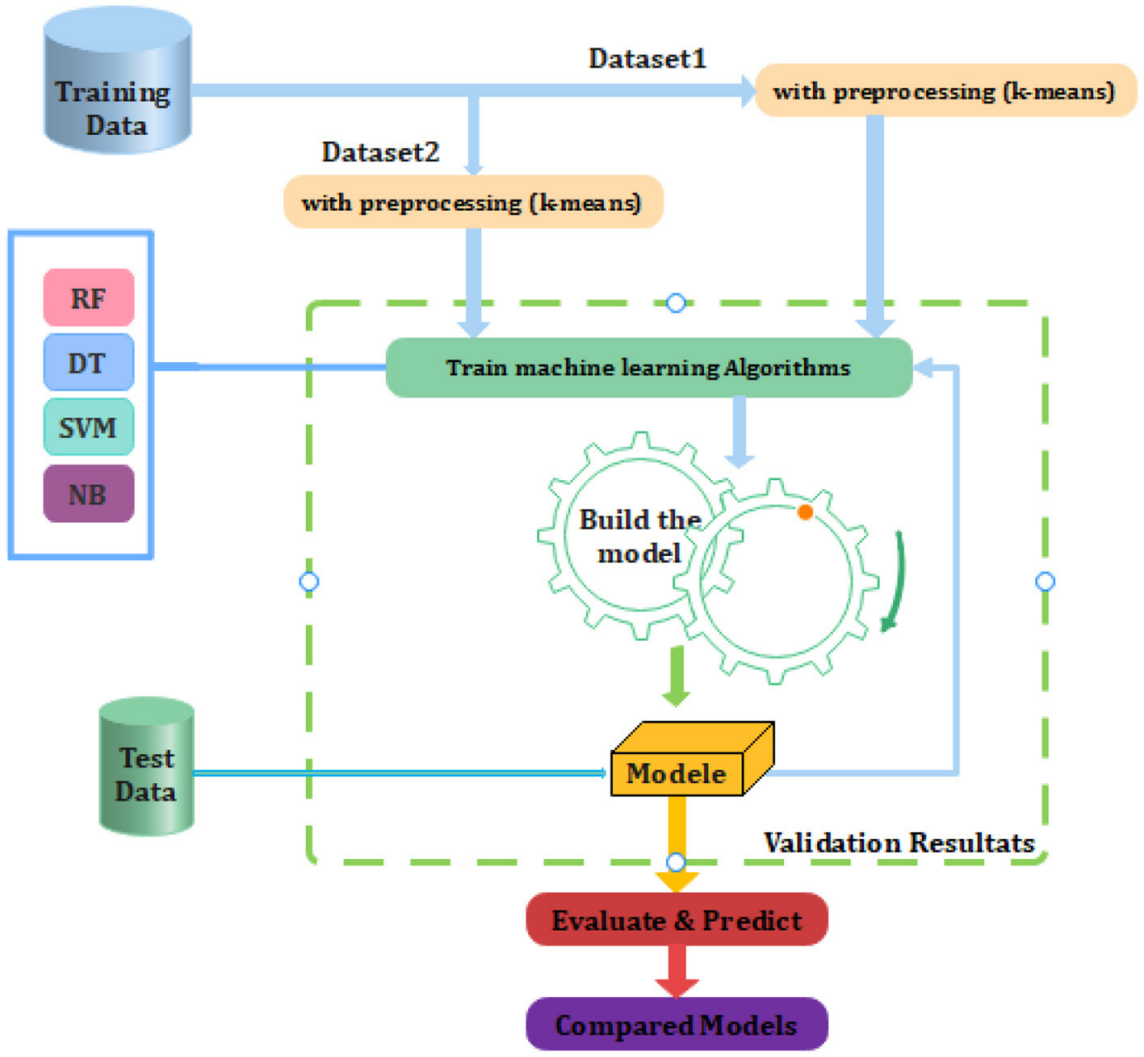
Proposed model diagram.

The proposed process is presented in the form of a model diagram in Figure 1 below. The following diagram depicts the flow of the research done in the process of building the model:

In this study, we split the data set into two parts: a training set and a test set. The training set is used to train the model. The test set is used to test the model and evaluate the accuracy.

In the second step we use a K-means algorithm for data correction in order to improve performance and control the classified model (By changing the number of clusters).

Next, we invite the learned algorithms to be tested using the test database. Finally model evaluation with other related work.

### Algorithms Used

#### Support Vector Machine

Support Vector Machine is based on statistical learning theory. SVMs were originally developed for binary classification, but can be effectively extended to multi-layer problems. SVM or Sequential Minimal Optimization (SMO) is a learning system that use a hypothesis space for linear functions in a high-dimensional space, and that has been trained using an optimization theory learning algorithm that employs a learning bias de-rived from statistical learning theory to achieve its results. SVM implements nonlinear class boundaries by translating nonlinear input vectors into a high-dimensional feature space using a linear model, which is implemented using the kernel of the SVM. Support vectors are training in-stances that are closer to the maximal hyper maximum level than the rest of the training examples. In order to define the binary layer boundaries support, all other training samples are rendered inapplicable.

As a result, the vectors are utilized to construct the ideal level hyper linear separation function (in the case of pattern recognition) or linear regression function (in the case of regression) in the feature space in question (13).

#### K-Means Clustering Algorithm

The k-means clustering algorithm is a machine learning algorithm that groups nearby points into clusters. In this algorithm, there is no learning model construction because we will locate the new point in any cluster based on its distance from all the clusters (mainly its distance from the cluster center or its arithmetic mean) and it is placed in the cluster that is closest. For example, imagine that you want to divide the points of a line into 3 groups. To Determine how close a point is to a particular group, we will use a measure of its distance from the group (for example, the distance between two points) (14).

#### Naive Bayes

NB is a classification approach in which the idea of independence and relatedness of all characteristics is defined as follows: Specifically, it specifies that the state of a given feature inside a class has no effect on the status of any other feature within the class. As a result of its foundation in conditional probability, it is regarded as a strong algorithm that may be utilized for classification applications. It performs effectively when dealing with data that has imbalance issues and missing values (15):


(1) $$\begin{array}{l} \text{P}\left(\text{A }|\text{ B}\right)=\frac{\text{P}\left(\text{B}|\text{A}\right)\text{P}\left(\text{A }\right)}{\text{P}\left(\text{B}\right)} \end{array}$$

- P(A|B): conditional probability that the response variable has a certain value given the input characteristics. Additionally, this is referred to as the posterior probability.
- P(A): The response variable's a priori probability.
- P(B): The likelihood that the training data or evidence is correct.
- P(B|A): This is referred to as the probability training data.

#### Decision Tree

DT learning is one of the predictive modeling techniques used in statistics, data mining, and machine learning. Use a decision tree (as a predictive model) to move from observations on an item (represented in the branches) to conclusions about the target value of the item (represented in the paper). They use a hierarchical representation the data structure in the form of a sequence decisions (tests) to predict an outcome or a category. Each individual (or observation), which must be allocated to a class, is represented by a collection of variables, which are tested in the nodes of the tree. In the internal nodes, testing is carried out, and choices are taken in the paper nodes (16). In graph theory, a tree is a linked graph that is undirected, acyclic, and has no edges. There are three categories of nodes:

- Root node: This is the base of the tree and the most sensitive element when the tree is created and before it is exploded.
- Internal node: refers to nodes that have offspring that are themselves nodes.
- Final nodes: that do not contain any branches.

There are many DT's algorithms, and we can cite: ID3, C4.5, CART, C5, CHAID, SLIQ, UFFT, VFDT... (16).

#### Random Forest

Random Forest algorithm (17) is for statistics and machines that employs several learning methods to improve prediction performance. The two-part algorithm A. Tree bagging b. Each tree is produced from tree bagging to random forest:

1. Sample N instances at random - but with replacement, from the original data if the number cases are N inside the training set. The training set for developing the tree will be this sample.
2. A random number of characteristics and the optimal division utilized for dividing the node are picked when there are M input variables. During the forest growth, the value M is kept constant.
3. Each tree is cultivated as much as possible.

### Dataset Used

We used two different databases in this study; Pima Indian Diabetes provided by the UCI Machine Learning repository (18) and a database extracted from the hospital in Frankfurt, Germany (19). Database extracted from the hospital in Frankfurt the first data is 2000 Pima Indian has 768 patient data with 8 attributes/features and one out-put with the patient's label/outcome (0: Not diabetic, 1: Diabetic). Two databases together consist some distinct medical variables, such as:

1. Pregnancies: number of pregnancies.
2. BMI: Body Mass Index (weight in kg / (height in m)^2^).
3. Insulin: Dose of insulin (mu U/ ml).
4. Age: Age at least 21 years.
5. Glucose: Plasma glucose concentration.
6. Blood Pressure: Diastolic blood pressure (mm Hg).
7. Skin Thickness: Thickness of the triceps skin fold (mm).
8. Diabetes Pedigree Function: Diabetes pedigree function (heredity).

And two classes (1 and 0)

- If class =1 implies diabetic patient.
- If class =0 implies non-diabetic patient.

The choice of these two bases is justified by the following criteria:

- The size of the database.
- Number of attributes.
- Number of classes.

#### Data Correction

Data cleaning is the next step in machine learning. It is considered one of the main steps in the working stages, and it is either building the model or breaking it. There is a saying that “the best data beats the most complex algorithms” in machine learning. Several aspects of data cleansing must be considered:

1. Discordances and omissions
2. Data mislabeling, same category repeated.
3. Invalid or missing data.
4. Outliers.

#### Unexpected Outliers in Both Bases

The observation and analysis of the two databases are presented in Table 1.

**Table 1:** The observation and analysis of the two databases.

**Variable**	**Observation and data analysis**	**Total number where the value is 0 (Pima base)**	**Total number where the value is 0 (Frankfort base)**
Blood pressure	The data shows that there are 0 number for blood pressure.	35	90
glucose	It is impossible for a person to have a glucose zero value even if they are fasting.	5	13
Skin thikness	For normal people, the thickness of the skin fold cannot be <10 mm.	227	573
BMI	It is impossible for a person to have a BMI 0 value.	11	28
Insulin	In a rare situation a person may have 0 insulin	374	956
Pregnancies	It is normal to have a zero value for this column so there is no need for cleaning.	111	301

#### Methods of Handling Invalid Data Values

##### The Existing Works

- Ignore/delete these cases: delete all the observations with zero values but in this method we get a significant loss of data (about 50% of the data set).
- Put the mean values: calculate the median value of a specific column and replace this value in that column where we have zero.
- Avoid using parameters: The model can avoid using parameters with too many incorrect values. This may help thicken skin, although it's hard to tell.

##### In This Study

- Use of a classification algorithm: use a classification algorithm to recover the missing data where we have zero and replace them with the value found.

In our case, we have to apply this method and we have chosen the k-means algorithm with a variable K number of cluster (group) and replacing each column needs cleaning by the representative of cluster.

### Evaluation Method

#### Train/Test

The data set is split into two parts: training and testing. The training set teaches the model. The test set is used to evaluate the model's correctness.

##### Pima Indian Database

“Test size = 0.2.” That is, 20% for the test and the rest 80% for the training.

##### Hospital Frankfurt Database

“Test size = 0.3.” That is, 30% for the test and the rest 70% for the training.

#### Accuracy Measures

This study uses Naive Bayes, Random Forest, SVM, and DT algorithms. Train/Test Split is used in experiments. This study uses Accuracy, F1-Measure, Recall, and Precision metrics to classify. See Table 2 for accuracy measures (20).

- True positives (TP), False positives (FP)
- True negatives (TN), False negatives (FN).

**Table 2:** Accuracy measures.

**Measures**	**Definitions**	**Formula**
Accuracy	Accuracy determines the accuracy of the algorithm in predicting instances	A = (TP + TN)/ (Nombre total d'échantillons)
Recall	Is the ability of a classification model to identify all relevant instances	R = TP/(TP + FN)
F1– Mesure	Is the weighted average of precision and recall	F = 2 * [(P * R)/(P + R)]
Precision	Classifiers correctness/accuracy is measured by Precision	P = TP/ (TP + FP)

### Experimental Results

This is clear from Table 3, which compares the different performance measures (accuracy, recall, and F1 score) used to evaluate the investigated machine learning models. Random Forest (RF) demonstrated the best accuracy when used in the tuned configuration (97.6 percent). In addition to Random Forest (RF), additional algorithms such as Decision Tree (DT) have demonstrated sufficient accuracy (97.5 percent). We can see that DT, Gaussian Naive Bayes, Random Forest, SVM, performed better. From the basic level, we can observe that Random Forest and DT work better than other algorithms (Table 4).

**Table 3:** Evaluation attributes results for different models (Frankfurt Germany).

**Algorithm used**	**Clusters**	**Accuracy**	**Recall score**	**F1 score**
Naïve bayes	C = 16	0.776	0.625	0.654
SVM	C = 20	0.783	0.566	0.638
Decision tree	C = 285	0.971	0.975	0.958
Random forest	C = 20	0.989	0.95	0.972

**Table 4:** Evaluation attributes results for the different models (Pima Indian).

**Algorithm used**	**Clusters**	**Accuracy**	**Recall score**	**F1 score**
Naïve bayes	C = 55	0.785	0.6	0.62
SVM	C = 16	0.831	0.533	0.648
Decision tree	C = 55	0.707	0.622	0.554
Random forest	C = 20	0.805	0.711	0.68

Based on different performance measures such as accuracy, recall, and F1 score, it is clear that the examined ML models are comparable; this is seen in the Table 5, SVM (Support Vector Machine) demonstrated the best accuracy when used in its optimized form (83.1 percent). Other algorithms, such as the Random Forest (RF), have demonstrated sufficient accuracy in addition to the Support Vector Machine (SVM) (80.5 percent). We can see that DT, Gaussian Naive Bayes, Random Forest, SVM, performed better. From the basic level, we can observe that Support Vector Machine and Random Forest work well than other algorithms.

**Table 5:** Comparison of the proposed work with the existing works (Pima Indian).

**Method**	**Accuracy**	**Reference**
Logistic regression	76.80%	(7)
Decision table	79.81%	(13)
Naïve Bayes	76.3%	(20)
Logistic Regression (LR)	80%	(10)
SVM	83.1%	Our study

From the results of this experimentation, we observe that the accuracy values for this database with all measurements are satisfactory with a disturbance sometimes the rate increases and sometimes it decreases by a small difference when changing the number of cluster as well as the random initialization of cluster centers can influence the results.

Note: After running the model several times, different results can be obtained in the same cluster number. This depends on the step of random initialization of the cluster centers.

According to the above table, the SVM model obtained the best accuracy which is equal to 83.1%. That is, among 153 attributes that were chosen for testing this model are classified 127 patients correctly. We select the SVM model as the most optimal model that works best for our dataset because it's high accuracy.

According to the above table, the Random Forest model obtained the best accuracy which is equal to 97.6%. That is, among 600 attributes that were chosen for testing this model are classified 582 patients correctly. We select the SVM model as the most optimal model that works best for our dataset because it's high accuracy.

## Discussion

Based on the results given in Tables 5, 6, the goal of the four algorithms is to better classify future observations while reducing classification errors. it can be concluded that the suggested models are more accurate than other type 2 diabetes prediction models that have been investigated in the research indicated in these tables. When comparing the above findings, it is obvious that the notion of utilizing the k-means algorithm was successful in our work; as a consequence, we infer that improving the quality of the data enhances the outcomes. This is consistent with other studies (26–35) which shows the predictive accuracy of machine learning algorithms.

**Table 6:** Comparison of the proposed work with the existing works (Frankfort Allemagne).

**Algorithmes used**	**Data correction methods**	**Train/test split size**	**Accuracy**	**Reference**
Random forest	The median	Test size = 0.2	91%	(21)
Gaussian process	The mean	Test size = 0.2	98.25%	(22)
DeepNN	Linear interpolation	Test size = 0.1	99.5%	(23–26)
Random forest	k-means	Test size = 0.2	100%	Our Study
Random forest	k-means	Test size = 0.3	97.6%	Our Study

## Conclusion

In this study, we proposed a supportive diagnosis system based on the comparison four models of prediction algorithms to predict diabetes in two different databases. On the basis of several performance assessment methodologies like as accuracy and recall, as well as the F1 score, different machine learning algorithms are compared and assessed. Using the classification results obtained, it can be concluded that the random forest machine learning technique provides more accurate prediction and higher performance than the other methods described in this study. However, when compared to other research accessible in the current literature, some of the other approaches utilized in this study, such as naive Bayes, DT and SVM, Random Forest, and others, produce the most optimum outcomes.

The main objective of this study is to help diabetologist to establish an accurate treatment routine for their diabetic patients. Due to the high accuracy and diagnose the disease in a shorter time and the rapid treatment, this study could open a window in the development of an electronic health system for diabetic patients. There are also a few aspects in this study that could be improved or expanded in the future. In perspective term:

- Creation of diabetes database for Algerian patients
- Diabetes prediction with the deep learning approach.
- Developed a solution based on an Android application in order to help people predict if they have diabetes.

## Data Availability Statement

The raw data supporting the conclusions of this article will be made available by the authors, without undue reservation.

## Author Contributions

ME: conceptualization, method, and analysis. OK: validation and conceptualization. ST: conceptualization and analysis. SG: methodology and investigation. NR-N: discussion and conclusion. CT: review, editing, and data curation. GA: results and conclusion. AL: editing and analysis. All authors contributed to the article and approved the submitted version.

## Funding

This research has been funded by the Research General Direction at Universidad Santiago de Cali under call No 01-2021.

## Conflict of Interest

The authors declare that the research was conducted in the absence of any commercial or financial relationships that could be construed as a potential conflict of interest.

## Publisher's Note

All claims expressed in this article are solely those of the authors and do not necessarily represent those of their affiliated organizations, or those of the publisher, the editors and the reviewers. Any product that may be evaluated in this article, or claim that may be made by its manufacturer, is not guaranteed or endorsed by the publisher.

## Abbreviations

UCI: University California Irvine
DT: Decision Trees
PIDD: Pima Indians Diabetes Database
WHO: World Health Organization
ML: Machine Learning
AI: Artificial Intelligence
FBG: Fasting Blood Glucose
SVM: Support Vector Machine
SMO: Sequential Minimal Optimization
KNN: K-Nearest Neighbors.
